# Effects of Light on Secondary Metabolite Biosynthesis in Medicinal Plants

**DOI:** 10.3389/fpls.2021.781236

**Published:** 2021-12-10

**Authors:** Shuncang Zhang, Lei Zhang, Haiyan Zou, Lin Qiu, Yuwei Zheng, Dongfeng Yang, Youping Wang

**Affiliations:** ^1^College of Bioscience and Biotechnology, Yangzhou University, Yangzhou, China; ^2^Key Laboratory of Plant Secondary Metabolism and Regulation of Zhejiang Province, College of Life Sciences and Medicine, Zhejiang Sci-Tech University, Hangzhou, China

**Keywords:** secondary metabolites, medicinal plants, light quality, light intensity, photoperiod

## Abstract

Secondary metabolites (SMs) found in medicinal plants are one of main sources of drugs, cosmetics, and health products. With the increase in demand for these bioactive compounds, improving the content and yield of SMs in medicinal plants has become increasingly important. The content and distribution of SMs in medicinal plants are closely related to environmental factors, especially light. In recent years, artificial light sources have been used in controlled environments for the production and conservation of medicinal germplasm. Therefore, it is essential to elucidate how light affects the accumulation of SMs in different plant species. Here, we systematically summarize recent advances in our understanding of the regulatory roles of light quality, light intensity, and photoperiod in the biosynthesis of three main types of SMs (polyphenols, alkaloids, and terpenoids), and the underlying mechanisms. This article provides a detailed overview of the role of light signaling pathways in SM biosynthesis, which will further promote the application of artificial light sources in medicinal plant production.

## Introduction

Medicinal plants play pivotal roles in human development and have been used from the prehistoric times to present day. According to the World Health Organization, 80% of the human population in developing countries relies on traditional medicines, mostly plant-based drugs, for primary healthcare needs. At present, at least 25% of the drugs worldwide are directly or indirectly derived from medicinal plants, which remain the main source of drugs. For example, artemisinin, derived from *Artemisia annua*, is widely used for treating malaria (Ansari et al., 2013). *Ajuga bracteosa* is a high-value medicinal plant that has been recommended as a treatment for gout rheumatism, paralysis, and amenorrhea (Rukh et al., 2019). Secondary metabolites (SMs) serve as the material basis of the clinically curative effects of medicinal plants. SMs refer to small molecular organic compounds not directly involved in plant growth and development, but are essential for the long-term survival of plants (Fraenkel, 1959; Demain and Fang, 2000). Besides their medicinal uses, SMs are also widely used in cosmetics and healthcare products (Craker and Gardner, 2006; Mathur and Velpandian, 2009; Schmidt, 2012).

Biosynthesis and accumulation of SMs in medicinal plants are affected by environmental factors, such as water, light, temperature, soil properties, and chemical stress (Verma and Shukla, 2015; Li et al., 2020). Among these factors, light is reported to affect the accumulation of almost all types of SMs. Light quality, light intensity, and photoperiod affect the SM content of plants. For example, the composition and content of SMs in the same plant species vary greatly among different regions because of the variation in light conditions (Huang and Liu, 2015). Controlled growing systems using artificial lighting have been widely applied with the increasing demand for natural products. These systems are initially developed for the production of out of season crops and vegetables. In recently years, they are also used to enhance the SMs yields in medicinal plants (Askari-Khorasgani and Pessarakli, 2019; Moon et al., 2020; Malik et al., 2021). Artificial lights take important place in controlled growing systems and light emitting diodes (LEDs) are supposed to be optimal artificial light sources at present time (Jung et al., 2021). Compared with other types of lamps, such as fluorescent, high-pressure sodium (HPS), and metal-halide, LEDs show equivalent or higher luminous efficacy, lack of radiant heat, and longer lifespan (Loi et al., 2021). Besides, LED can also produce the monochromatic light wavelength and make it more convenient to change the light quality constitution in the controlled growing systems (Palmitessa et al., 2021). Understanding how light affects SM biosynthesis is essential for the cultivation of medicinal plants in a controlled environment as well as under open field conditions. In this review, we discuss the roles of light in the accumulation of different types of SMs, with the aim to identify the gaps in research and to provide a reference for the further investigation of the mechanism underlying light-mediated SM biosynthesis in medicinal plants.

## Main Secondary Metabolites in Medicinal Plants

**Figure 1:**
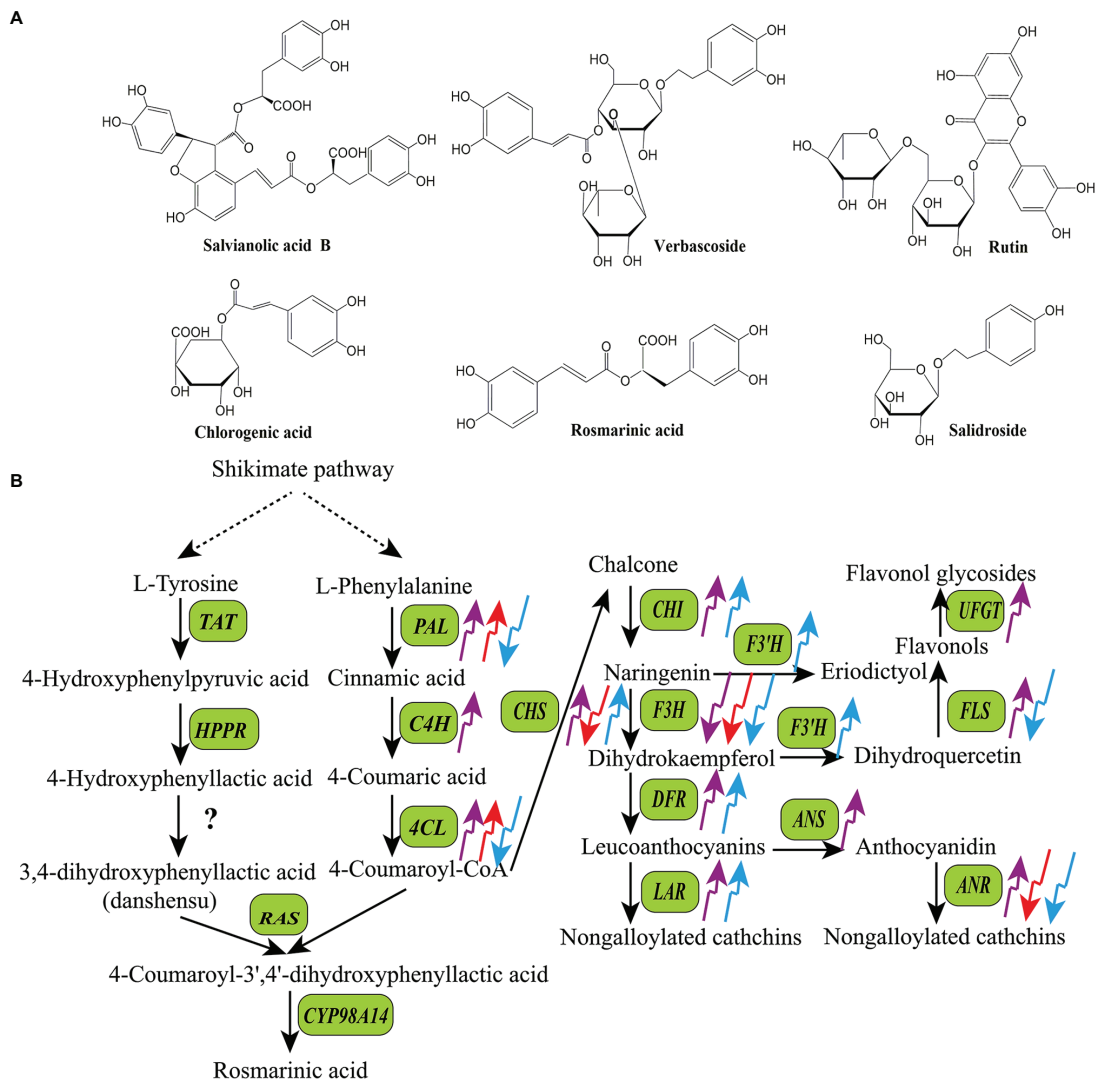
Structures of some bioactive polyphenols in medicinal plants **(A)** and the effects of light quality on the transcripts of key enzyme genes in the biosynthetic pathways of polyphenols **(B)**. The upward arrows indicate upregulation and the downward arrows indicate downregulation. The purple arrow, red arrow, and blue arrow indicate UV-B, red light, and blue light, respectively. *ANR*, anthocyanidin reductase; *ANS*, anthocyanidin synthase; *CHS*, chalcone synthase; *CHI*, chalcone isomerase; *CYP98A14*, cytochrome p450 98A14; *C4H*, cinnamic acid 4-hydroxylase; *DFR*, dihydroflavonol 4-reductase; *FLS*, flavonol synthase; *F3′H*, flavonol 3′-hydroxylase; *F3H*, flavanone 3-hydroxylase; *HPPR*, 4-hydroxyphenylpyruvate reductase; *LAR*, leucoanthocyanidin reductase; *PAL*, phenylalanine ammonia-lyase; *RAS*, rosmarinic acid synthase; *TAT*, tyrosine aminotransferase; *UFGT*, UDP flavonoid glucosyltransferase; *4CL*, 4-coumaric acid: CoA ligase.

Based on their structures and biosynthetic pathways, plant SMs are mainly divided into polyphenols (phenolics), terpenoids, and alkaloids (Chiocchio et al., 2021). Polyphenols are a large and complex family of phytochemicals containing at least one aromatic ring and a hydroxyl group as functional derivatives. Over 8,000 polyphenols have been identified in plants to date. They are present in almost all plant species and have gained considerable attention because of their nutritional and pharmaceutical applications (Balasundram et al., 2006). According to the biosynthetic pathways, basic skeletons, and hydroxyl groups, polyphenols are categorized into different sub-classes, including coumarins, lignans, phenolic acids, flavonoids, and tannins (Huang and Liu, 2015; Chiocchio et al., 2021). Coumarins (C_6_-C_3_) are a class of lactones structurally constructed by a benzene ring fused to α-pyrone ring, such as aesculin, cnidium lactone, and alpha-Angelica lactone. Lignans [(C_6_-C_3_)_2_] are phenolic dimers with a 2,3-dibenzylbutane skeleton, such as phyllanthin, arctiin, and podophyllotoxin. Phenolic acids present compounds containing a carboxylic group among the substituents on the benzene ring, including benzoic acid derivates (C_6_-C_1_) and hydroxycinnamic derivates (C_6_-C_3_). Flavonoids (C_6_-C_3_-C_6_) refer to compounds consisting of two benzene rings linked by a short three carbon chain, such as chalcones, flavones, flavonols, dihydroflavones, dihydroflavonols, isoflavones, and dihydroisoflavones. Tannins are high molecular polyphenols polymerized by flavonoids units (condensed tannins) or gallic acid esterified with monosaccharide (hydrolysable tannins; King and Young, 1999). Figure 1A shows structures of some common bioactive polyphenols in medicinal plants, including three phenolic acids (salvianolic acid B, chlorogenic acid, and rosmarinic acid), two phenylethanol glycosides (verbascoside and salidroside), and one flavanone derivate (rutin). Polyphenols are biosynthesized *via* phenylpropanoid pathway in plants, they share a common upstream biosynthetic pathway derived from the shikimic acid pathway, but the downstream biosynthetic pathways of different polyphenols are distinct (Figure 1B; Dixon and Paiva, 1995).

**Figure 2:**
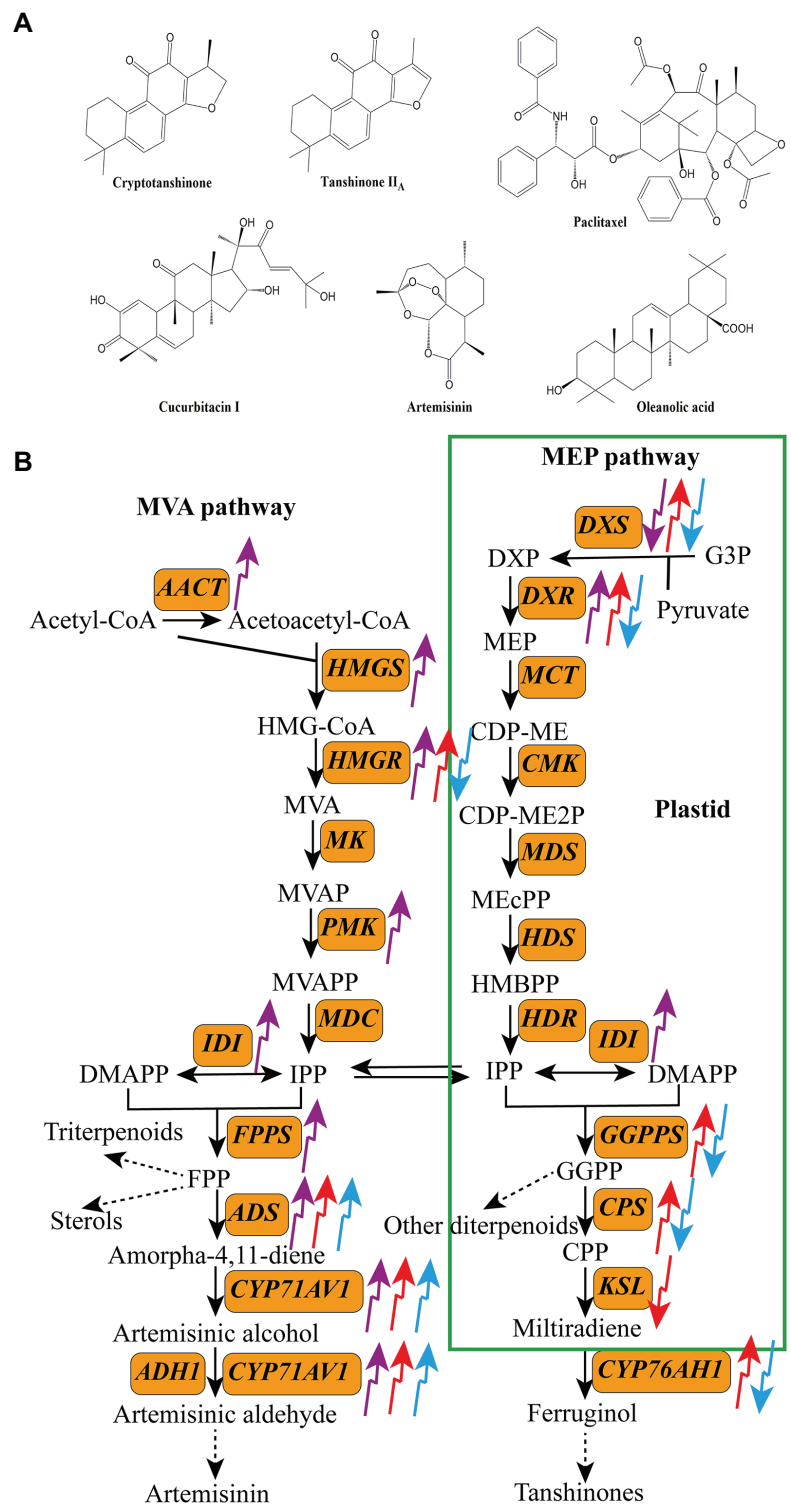
Structures of some bioactive terpenoids in medicinal plants **(A)** and the effects of light quality on the transcripts of key enzyme genes in the biosynthetic pathways of terpenoids **(B)**. The upward arrows indicate upregulation and the downward arrows indicate downregulation. The purple arrow, red arrow, and blue arrow indicate UV-B, red light, and blue light, respectively. *AACT*, acetyl-CoA acyltransferase; *ADH1*, alcohol dehydrogenase 1; *ADS*, amorpha-4,11-diene synthase; *CMK*, 4-diphosphocytidyl-2C-methyl-D-erythritol kinase; *CPS*, copalyl diphosphate synthase; *CYP71AV1*, cytochrome P450 71AV1; *CYP76AH1*, miltiradiene oxidase; *DXR*, 1-deoxy-D-lxylulose-5-phosphate reductoisomerase; *DXS*, 1-deoxy-D-xylulose-5-phosphate synthase; *FPPS*, farnesyl diphosphate synthase; *GGPPS*, geranylgeranyl diphosphate synthase; *HDR*, hydroxy-2-methyl-2-(E)-butenyl 4-diphosphate reductase; *HDS*, hydroxymethylbutenyl diphosphate synthase; *HMGR*, 3-hydroxy-3-methylglutaryl CoA reductase; *HMGS*, 3-hydroxy-3-methylglutary-1 CoA synthase; *IDI*, isopentenyl diphosphate isomerase; *KSL*, kaurene synthase-like; *MCT*, 2-C-methyl-D-erythritol-4-phosphate cytidylyltransferase; *MDC*, mevalonate 5-diphosphate decarboxylase; *MDS*, 2-C-methyl-D-erythritol-2,4-cyclodiphosphate synthase; *MK*, mevalonate kinase; *PMK*, phosphomevalonate kinase.

Terpenoids are compounds with isoprene as the structural unit. According to the number of isoprene structural units, terpenoids are divided into five categories: monoterpenes, sesquiterpenes, diterpenes, triterpenes, and tetraterpenes (Bohlmann et al., 1998). Figure 2A shows structures of some common bioactive terpenoids in medicinal plants, including one sesquiterpene (artemisinin), three diterpenes (cryptotanshinone, tanshinone II_A_, and paclitaxel), and two triterpenes (cucurbitacin I and oleanolic acid). Terpenoids are synthesized *via* two parallel upstream pathways: the mevalonate pathway (MVA) and methylerythritol-4-phosphate pathway (MEP; da Silva et al., 2017). Acetyl-CoA acts as the precursor of terpenoids in the MVA pathway, whereas pyruvate and glyceraldehyde-3-phosphate (G3P) serve as precursors in the MEP pathway. These precursors are converted to isopentenyl pyrophosphate (IPP) through a series of enzymatic reactions, which is then converted to its isomer dimethyl-allyl pyrophosphate (DMAPP) by isopentenyl diphosphate isomerase (IDI). Both DMAPP and IPP are converted to geranylgeranyl diphosphate (GGPP) by geranylgeranyl diphosphate synthase (GGPPS), and then, GGPP is used to produce diterpenes, tetraterpenes, and their derivatives (Newman and Chappell, 1999). DMAPP and IPP are also converted to farnesyl diphosphate (FPP) and geranyl diphosphate (GPP) by farnesyl diphosphate synthase (FPPS) and geranyl diphosphate synthase (GPPS), respectively. Both FPP and GPP then act as precursors of monoterpenes, sesquiterpenes, and triterpenes (Figure 2B; Chappell, 1995; Bergman et al., 2019).

**Figure 3:**
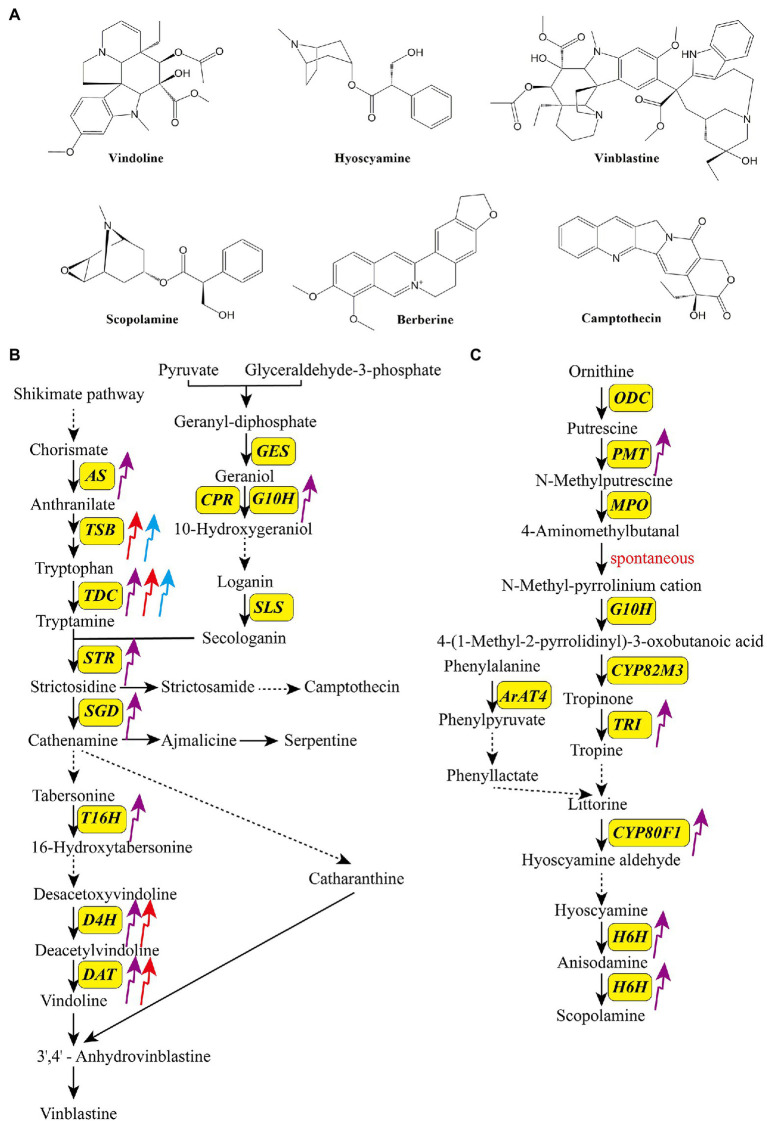
Structures of some bioactive alkaloids in medicinal plants **(A)** and the effects of light quality on the transcripts of key enzyme genes in the biosynthetic pathways of monoterpenoid indole terpenoid alkaloids **(B)** and tropane alkaloids **(C)**. The upward arrows indicate upregulation and the downward arrows indicate downregulation. The purple arrow, red arrow, and blue arrow indicate UV-B, red light, and blue light, respectively. *ArAT4*, aromatic amino acid aminotransferase 4; *AS*, anthranilate synthase; *CPR*, cytochrome P450 reductase; *CYP80F1*, cytochrome P450 80F1; *CYP80M3*, cytochrome P450 80 M3; *DAT*, 6-17-O-deacetylvindoline O-acetyltransferase; *D4H*, deacetoxyvindoline 4-hydroxylase; *GES*, geraniol synthase; *H6H*, hyoscyamine 6b-hydroxylase; *G10H*, geraniol-10-hydroxylase; *MPO*, N-methylputrescine oxidase; *ODC*, ornithine decarboxylase; *PMT*, putrescine N-methyltransferase; *SGD*, strictosidine β-glucosidase; *SLS*, secologanin synthase; *STR*, strictosidine synthase; *TDC*, tryptophan decarboxylase; *TRI*, tropinone reductase I; *T16H*, tabersonine 16-hydroxylase.

Alkaloids form a large class of heterocyclic nitrogen organic compounds, with over 10,000 isolated to date. Given their antitumor, antibacterial, and anti-inflammatory activities, alkaloids have been widely used for the production of medications (Jain et al., 2019; Zhang et al., 2021). Depending on their biosynthetic pathways and chemical structures, alkaloids are classified into five groups: terpene indole, benzylisoquinoline, tropine, purine, and pyrrolizidine alkaloids (Bhambhani et al., 2021). Figure 3A shows structures of some common bioactive alkaloids in medicinal plants, including three monoterpenoid indole alkaloids (MIAs; vindoline, vinblastine, and camptothecin), one benzylisoquinoline alkaloid (berberine), and two tropine alkaloids (hyoscyamine and scopolamine). The accumulations of MIAs and tropane alkaloids have been reported to be affected by light. Biosynthetic pathways of MIAs and tropane alkaloids have been characterized. MIAs are synthesized *via* two parallel upstream pathways, generating tryptamine and secologanin, which are converted to strictosidine by strictosidine synthase (STR), and then to various MIAs, such as camptothecin, serpentine, and vinblastine (Herrmann, 1995; Radwanski et al., 1996; Huang et al., 2016). Ornithine and phenylalanine, the initial precursors of tropine alkaloids, are converted to littorine, which is then converted to anisodamine and scopolamine *via* several enzymatic reactions (Figures 3B,C; Nguyen et al., 2015; Qiu et al., 2020).

## Light Quality

### Light Spectra and Photoreceptors

Depending on the light wavelength, the solar spectrum is divided into three parts, namely ultraviolet light (200–400nm; UV-A, 315–400nm; UV-B, 280–320nm; UV-C, 200–280nm), visible light or photosynthetically active radiation (PAR; blue light, 400–500nm; green light, 500–600nm; red light, 600–700nm), and far-red light (700–800nm). Photoreceptors are indispensable for light sensing and light signal transduction in plants. To date, at least five kinds of photoreceptors have been identified in the model plant species *Arabidopsis thaliana*: (1) phytochromes (phyA–phyE), which mainly perceive red light and far-red light (Sharrock and Quail, 1989; Sullivan and Deng, 2003; Sanchez et al., 2020); (2) cryptochromes (crys), which mainly perceive blue light and UV-A. (3) phototropins (phots), which mainly perceive blue light (Gyula et al., 2003); (4) zeitlupes, which mainly perceive blue light and green light (450–520nm; Somers et al., 2000); and (5) UV photoreceptor UVR8, which mainly perceives UV-B (280–315nm; Rizzini et al., 2011; Christie et al., 2012). Among the five phytochromes, phyA is the main photoreceptor that perceives far-red light, while phyB mainly perceives red light. Owing to these photoreceptors, plants can accurately detect changes in light wavelength, direction, intensity, and duration, and respond timely.

### Polyphenols

The production of polyphenols is significantly affected by light in medicinal plants. UV-B is an important part of the solar spectrum; however, excessive UV-B can damage plants. To deal with UV-B-induced stress and to improve their resilience to adverse conditions, plants synthesize various types of SMs (Schreiner et al., 2012; Kumari and Prasad, 2013; Takshak and Agrawal, 2019). Among these SMs, polyphenols, owing to their high antioxidant potential, play important roles in the acclimation of plants to UV-B (Takshak and Agrawal, 2019). The biosynthesis of bioactive polyphenols is affected by UV-B in many medicinal plants (Table 1; Rai et al., 2011; Pandey and Pandey-Rai, 2014; Takshak and Agrawal, 2014; Hamid et al., 2019). Takshak and Agrawal (2015) showed that anthocyanin contents of *Coleus forskohlii* plants treated with supplementary UV-B (ambient +0.042 Wm^−2^) were 1.34-, 1.67-, and 1.96-fold higher than that in control at 40, 70, and 100 days after transplantation (DAT), respectively. Zhang et al. (2017) investigated the effects of UV-B on SM accumulation in *Prunella vulgaris* and showed that UV-B radiation significantly increased the contents of total flavonoids, rosmarinic acid, caffeic acid, and hyperoside. In *Rosmarinus officinalis* plants treated with UV-B (0.359 Wm^−2^), the contents of rosmarinic acid and carnosic acid were 2.34- and 1.78-fold higher, respectively, than that of the control (Luis et al., 2007). The total phenolic content of *Cymbopogon citratus* increased by 45% after the UV-B treatment (Kumari and Agrawal, 2010). In addition to UV-B, UV-A also affects the production of polyphenols in some medicinal plants. For example, in *Crepis japonica*, treatment with UV-A (6–25 W m^−2^) significantly promoted the production of caffeic acid and chlorogenic acid (Constantino et al., 2017). In another study, a 3-day UV-A treatment of *Ixeris dentata* increased the total phenolic and total flavonoid contents by 60 and 40%, respectively, compared with the control (Lee et al., 2013).

**Table 1:** The effects of light quality on the accumulation of phytochemicals in some medicinal plants.

Species	Class	Light quality	Treatment time	Modulation	References
*Artemisia annua*	Polyphenol	UV-B (2.8 W m^−2^)	1, 2, 3 and 4 h	↑Flavonoids ↑Anthocyanin	Pandey and Pandey-Rai, 2014
*Cymbopogon citratus*	Polyphenol	UV-B (ambient+0.021 and+0.042 W m^−2^)	3 h d^−1^ for 80 days	↑Flavonoids	Kumari and Agrawal, 2010
*Fagopyrum esculentum*	Polyphenol	UV-B (890 W m^−2^)	24 h	↑Anthocyanin ↑Rutin	Tsurunaga et al., 2013
*Heteropogon contortus*	Polyphenol	UV-B (ambient+0.083 W m^−2^)	3 h, d^−1^ for 110 days	↑Tannins ↑Total phenolics	Hamid et al., 2019
*Perilla frutescens*	Polyphenol	UV-B (0.05 W m^−2^)	3 h	↑Rosmarinic acid	Yoshida et al., 2021
*Prunella vulgaris*	Polyphenol	UV-B (0.35 W m^−2^)	30 min d^−1^ for 15 days	↑Total flavonoids ↑Rosmarinic acid ↑Caffeic acid	Zhang et al., 2017
*Rosmarinus officinalis*	Polyphenol	UV-B (0.063 and 0.359 W m^−2^)	14 days	↑Rosmarinic acid ↑Carnosic acid ↑Total phenolics	Luis et al., 2007
*Salvia verticillata*	Polyphenol	UV-B (0.127 W m^−2^)	1 h, d^−1^ for 5 days	↑Chlorogenic acid ↑Caffeic acid ↑Rosmarinic acid	Rizi et al., 2021
*Tropaeolum majus*	Polyphenol	UV-B (7.5e-4 and 1.5e-3 W m^−2^)	2 and 22 h	↑Glucotropaeolin	Schreiner et al., 2009
*Withania somnifera*	Polyphenol	UV-B (0.111 W m^−2^)	3 h d^−1^ for 100 days	↑Anthocyanins, ↑Flavonoids	Takshak and Agrawal, 2014
*Crepis japonica*	Polyphenol	UV-A (6–25 W m^−2^)	11.5 h d^−1^ for 60 days	↑Caffeic acid ↑Chlorogenic acid	Constantino et al., 2017
*Ixeris dentata*	Polyphenol	UV-A UV-B	24 h·d^−1^ for 5 days 4 h·d^−1^ for 3 days	↑Total phenolics ↑Total flavonoid ↑Total phenolics	Lee et al., 2013
*Cichorium intybus*	Polyphenol	Blue light followed by white light (25 μmol m^−2^ s^−1^ and 320 μmol m^−2^ s^−1^, respectively)	14 h·d^−1^ for 30 days (blue light), 14 h·d^−1^ for 27 days (white light)	↓Chlorogenic acid ↓Caftaric acid ↑Kaempferol-3-(6″-acetyl)-glucoside ↓Isorhamnetin-3-(6″-acetyl)-glucoside ↑Isorhamnetin-3-glucoside ↓Chlorogenic acid	Sytar et al., 2019
		Orange light followed by white light (40 μmol m^−2^ s^−1^ and 320 μmol m^−2^ s^−1^, respectively)	14 h·d^−1^ for 30 days (orange light), 14 h·d^−1^ for 27 days (white light)	↓Kaempferol-3-(6″-acetyl)-glucoside ↑Isorhamnetin-3-(6″-acetyl)-glucoside ↑Isorhamnetin-3-glucoside ↑Feruloyl-malate ↓Chlorogenic acid ↑Quercetin-3-(6″-acetyl)-glucoside	
		Red light followed by white light (40 μmol m^−2^ s^−1^ and 320 μmol m^−2^ s^−1^, respectively)	14 h·d^−1^ for 30 days (red light), 14 h·d^−1^ for 27 days (white light)	↓Isorhamnetin-3-glucoside	
*Dracocephalum forrestii*	Polyphenol	Blue light (40 μmol m^−2^ s^−1^)	16 h d^−1^ for 35 days	↑Chlorogenic acid ↑Salvianolic acid B ↓Rosmarinic acid	Weremczuk-Jezyna et al., 2021
*Drosera indica*	Polyphenol	Blue light (115 μmol m^−2^ s^−1^)	16 h d^−1^ for 14 days	↑Plumbagin	Boonsnongcheep et al., 2019
*F. esculentum*	Polyphenol	Blue light	16 h d^−1^ for 7 days	↑C-glycosylflavones ↑Orientin ↑Vitexin and its isomers ↑Rutin	Nam et al., 2018
*Ocimum basilicum*	Polyphenol	Blue light (40–50 μmol m^−2^ s^−1^)	24 h d^−1^ for 35 days	↑Total phenolics ↑Total flavonoids	Nazir et al., 2020
*Rhodiola imbricata*	Polyphenol	Blue light (25–27 μmol m^−2^ s^−1^)	24 h·d^−1^ for 30 days	↑Total phenolics ↑Total flavonoids ↑Salidroside	Kapoor et al., 2018
		Red light (35–37 μmol m^−2^ s^−1^)	24 h·d^−1^ for 30 days	↑Salidroside	
*Salvia przewalskii*	Polyphenol	Blue light (40 μmol m^−2^ s^−1^)	16 h d^−1^ for 12 days	↑Salvianolic acid A ↑Salvianolic acid B ↑Rosmarinic acid ↓Protocatechualdehyde	Li, 2014
		Red light (40 μmol m^−2^ s^−1^)	16 h d^−1^ for 12 days	↑Salvianolic acid A ↑Salvianolic acid B ↑Rosmarinic acid ↑Protocatechualdehyde	
*Sarcandra glabra*	Polyphenol	Blue and red light (80 μmol m^−2^ s^−1^)	16 h d^−1^ for 60 days	↓Isofraxidin ↓Scopoletin ↓Rosmarinic acid	Xie et al., 2021
*Scutellaria baicalensis*	Polyphenol	Blue and red light (80 μmol m^−2^ s^−1^)	30 days	↑Baicalin ↑Baicalein ↑Wogonin	Stepanova et al., 2020
*Verbena officinalis*	Polyphenol	Blue light (40 μmol m^−2^ s^−1^)	16 h d^−1^ for 28 days	↑Verbascoside ↑Isoverbascoside	Kubica et al., 2020
*Achyranthes bidentata*	Terpenoid	UV-B (0.205 W m^−2^)	2 h and 3 h	↑Oleanolic acid ↑Ecdysterone	Li et al., 2019
*A. annua*	Terpenoid	UV-B (0.047 W m^−2^)	30 min d^−1^ for 14 days	↑Artemisinin	Rai et al., 2011
		UV-C (0.066 W m^−2^)	30 min d^−1^ for 14 days	↑Artemisinin	
*A. annua*	Terpenoid	UV-B (0.017 W m^−2^)	1 h d^−1^ for 10 days	↑Artemisinin	Pan et al., 2014
*Salvia miltiorrhiza*	Terpenoid	UV-B (0.4 W m^−2^)	40 min	↑Total tanshinones ↑Cryptotanshinone ↑Tanshinone II_A_ ↑Tanshinone I	Wang et al., 2016
*A. annua*	Terpenoid	Blue and red light (50±5 μmol m^−2^ s^−1^)	24 h d^−1^ for 2 days	↑Artemisinin	Zhang et al., 2018b
*Dysphania ambrosioides*	Terpenoid	Blue light (60 μmol m^−2^ s^−1^)	16 h d^−1^ for 40 days	↓Z-ascaridole	de Carvalho et al., 2020
*Mentha canadensis*	Terpenoid	Blue and red light (6.7 and 7.1 μmol m^−2^ s^−1^, respectively)	6 h d^−1^ for 14 days	↑Pulegone ↑Menthofuran ↑Menthol	Ueda et al., 2021
*Perovskia atriplicifolia*	Terpenoid	Blue and red light (300 μmol m^−2^ s^−1^)	16 h d^−1^ for 60 days	↓α-Pinene ↑Camphene ↑δ-3-Carene ↓Camphor	Ghaffari et al., 2019
*S. przewalskii*	Terpenoid	Blue and red light (40 μmol m^−2^ s^−1^)	16 h d^−1^ for 12 days	↑Dihydrotanshinone ↑Cryptotanshinone ↑Tanshinone II_A_ ↓Tanshinone I	Li, 2014
*Aquilaria agallocha*	Terpenoid	Red light (~15 μmol m^−2^ s^−1^)	24 h d^−1^ for 2 days	↑Cucurbitacin I ↑Cucurbitacin E	Kuo et al., 2015
		Far-red light (~15 μmol m^−2^ s^−1^)	24 h d^−1^ for 5 days	↓Cucurbitacin I ↓Cucurbitacin E	
*Catharanthus roseus*	Alkaloid	UV-B (13.45 W m^−2^)	1 h	↑Strictosidine ↑Vindoline ↑Catharanthine ↑Ajmalicine	Zhu et al., 2015
*Clematis terniflora*	Alkaloid	UV-B (1.208 W m^−2^)	5 h	↑(6-Hydroxyl-1H-indol-3-yl) carboxylic acid methyl ester	Gao et al., 2016
*Mahonia bealei*	Alkaloid	UV-B (1.208 W m^−2^)	6 h	↓Berberine ↓Palmatine ↑Columbamine	Zhang et al., 2014
*W. somnifera*	Alkaloid	UV-B (ambient +0.042 W m^−2^)	3 h d^−1^ for 100 days	↑Total alkaloids ↓Withanolide A	Takshak and Agrawal, 2014
*Camptotheca acuminata*	Alkaloid	Blue light (1,200±50 μmol m^−2^ s-^1^)	12 h d^−1^ for 45 days	↑Camptothecin	Liu et al., 2015
*Psychotria leiocarpa*	Alkaloid	Blue, red and far-red light (30 μmol m^−2^s^−1^)	20 d	↑N,-ᵝ-ᶛ-glucopyranosyl vincosamide	Matsuura et al., 2016
*C. roseus*	Alkaloid	Red light (150μmolm^−2^s^−1^)	16h d^−1^ for 28days	↑Vindoline ↑Catharanthine	Ohashi et al., 2013

*The upward arrow in the table represents the increased content of the corresponding phytochemical, the downward arrow in the table represents the decreased content of the corresponding phytochemical*.

The mechanism underlying the regulatory effects of UV-B on the synthesis of polyphenols is not fully understood. However, it has been confirmed that UV-B affects the activities of key polyphenol biosynthetic enzymes and/or transcript levels of the corresponding genes in many medicinal plants (Figure 1B). For example, supplemental UV-B radiation significantly improved the activities of phenylalanine ammonia-lyase (PAL), cinnamyl alcohol dehydrogenase (CAD), 4-coumaric acid: CoA ligase (4CL), CHI, and dihydroflavonol 4-reductase (DFR), and enhanced the contents of flavonoids and phenolicp0s in the leaves of *C. forskohlii* and *Withania somnifera* (Takshak and Agrawal, 2014, 2015). In *Sinopodophyllum hexandrum*, treatment with UV-B (1.07 W·m^−2^) significantly decreased the content of podophyllotoxin as well as transcript levels of 12 related genes, including *cinnamic acid 3-hydroxylase* (*C3H*), *caffeoyl-CoA O-methyltransferase* (*CCoAMT*), *cinnamoyl-CoA reductase* (*CCR*), *CAD*, *dirigent protein oxidase* (*DPO*), *pinoresinol-lariciresinol reductase* (*PLR*), *secoisolariciresinol dehydrogenase* (*SDH*), *cytochrome P450 719A23* (*CPY719A23*), *O-methyltransferase3* (*OMT3*), *cytochrome P450 71CU1* (*CYP71CU1*), *OMT1*, and *2-oxoglutarate/Fe(II)-dependent dioxygenase* (*2-ODD*; Lv et al., 2021). In *Glycyrrhiza uralensis*, Zhang et al. (2018a) showed that UV-B radiation stimulated the expression of several genes involved in the flavonoid biosynthetic pathway, such as *cinnamic acid 4-hydroxylase* (*C4H*), *PAL*, *CHS*, *CHI*, and *FLS*. Wulff et al. (1999) reported that UV-B stimulated the accumulation of quercetin-3-glycoside and increased the expression of *CHS* in *Betula pendula*.

Blue and red light wavelengths are two important light qualities involved in plant growth and development. Both light qualities are widely recognized as effective elicitors that regulate the accumulation of bioactive compounds in medicinal plants (Table 1; Dou et al., 2017). Fazal et al. (2016a) investigated the effects of monochromatic blue, green, yellow, and red light wavelengths on the production of polyphenols in *P. vulgaris* calli, and found that the calli accumulated most total phenolics (23.9 mg g^−1^ DW) and total flavonoids (1.65 mg g^−1^ DW) under blue light. Similarly, Kapoor et al. (2018) revealed that the callus cultures of *Rhodiola imbricata* accumulated the highest amounts of salidroside, total phenolics, and total flavonoids under blue light compared with those under red light, green light, RGB (40% red:40% green:20% blue), and white light. Kubica et al. (2020) found that both blue light and red light significantly stimulated the accumulation of verbascoside compared with fluorescent lamps (control) in *Verbena officinalis*. Coumarins is an important class of phenols in medicinal plants. The biosynthesis and accumulation of coumarins are significantly affected by blue and red light. For instance, Xie et al. (2021) treated *Sarcandra glabra* seedlings with different monochromatic lights for 60 days and found that the content of fraxetin and 6-methylcoumarin in red light treated group was 45 and 16% of that in control (under white light), respectively. The content of these two coumarins in blue light treated group was 51 and 11% of that in control, respectively. Khurshid et al. (2020) revealed that blue and red light stimulated the accumulation of coumarins in callus culture of *Eclipta alba*. The content of coumarin, wedelolactone, and demethylwedelolactone in the red light treated group (40–50 μmol m^−2^ s^−1^, 28 d) was 3.07-, 1.59-, and 1.59-fold of that in control (white light), respectively. The content of these compounds in the blue light treated group was 2.24-, 1.43-, and 1.29-fold of that in control, respectively. Combined blue and red light are often used to improve the growth and SM content of medicinal plants simultaneously. For example, Lobiuc et al. (2017) cultured *Ocimum basilicum* seedlings under different light conditions, and found that the dry mass, rosmarinic acid, and caffeic acid contents of seedlings were 1.45-, 15-, and 4-fold higher under combined red and blue light (1R:2B), respectively, than under control (white) conditions. Zhang et al. (2020) treated *Salvia miltiorrhiza* seedlings with monochromatic blue light (B), monochromatic red light (R), and combined blue and red light, and showed that seedling growth and phenolic acid production were stimulated under 7R:3B. Wei et al. (2021) found that combined red and blue LED light (1.61R:1B) improved the growth and cannabidiol content of *Cannabis sativa* seedlings, and increased the aboveground plant biomass, flower biomass, and flower cannabidiol content by 15.2, 238, and 36.53%, respectively, compared with the control (Seedlings growth under high-pressure sodium light). Silva et al. (2020) compared the effects of different light qualities on morphogenesis and SM production in *Pfaffia glomerata* and found that equal proportion of red and blue light (1R:1B) was the best light condition for the accumulation of biomass, anthocyanins, and 20-hydroxyecdisone.

Similar to UV-B, blue and red light wavelengths affect the production of phenylpropanoids by regulating the transcript levels of phenylpropanoid biosynthetic genes (Figure 1B; Hao et al., 2016; Alrifai et al., 2019). For example, Zhang et al. (2019b) found that the expression levels of *PAL* and *4CL*, which are required for phlorizin synthesis, are correlated with phlorizin content under red and blue light in *Lithocarpus polystachyus*. Liu et al. (2018a) reported that blue light dramatically induced flavonoid biosynthesis in *Cyclocarya paliurus* leaves, and the flavonoid content was positively correlated with the transcript levels of *PAL*, *4CL*, and *CHS*. MYB transcription factors and microRNAs are also involved in light-induced polyphenol biosynthesis in some medicinal plants. For example, in *Fagopyrum tataricum*, FtMYB16 directly binds to the promoter region of the *flavanone 3-hydroxylase* (*F3′H*) gene under red and bule light to induce its expression and enhance the flavonoid content (Zhang et al., 2019a). In *Dimocarpus longan*, miR393, miR394, and miR395 act as positive regulators of epicatechin production under blue light (Li et al., 2018b).

### Terpenoids

Biosynthesis of terpenoids in medicinal plants is closely related to light conditions (Figure 2B). Both UV and visible light act as important elicitors of terpenoid synthesis (Table 1; Zhang and Bjorn, 2009; Kawoosa et al., 2010; Xie et al., 2021). Among the different light qualities, UV-B is reported to promote the accumulation of terpenoids in many plant species (Takshak and Agrawal, 2019). For instance, in *C. citratus*, treatment with supplemental UV-B increased the total essential oil yield by 25.7% (Kumari and Agrawal, 2010). Artemisinin is a typical sesquiterpene lactone that has attracted considerable attention because of its widespread application in malaria treatment (Ansari et al., 2013). Several studies showed that UV-B treatment induces the biosynthesis of artemisinin and enhances the expression levels of key enzyme-encoding genes, such as *3-hydroxy-3-methylglutaryl CoA reductase* (*HMGR*), *1-deoxy-D-xylulose-5-phosphate reductoisomerase* (*DXR*), *isopentenyl pyrophosphate isomerase* (*IPPi*), *FPS*, *amorpha-4, 11-diene synthase* (*ADS*) gene, *cytochrome P450 71AV1* (*CYP71AV1*), and dihydroartemisinic aldehyde reductase (*RED1*; Yin et al., 2008; Rai et al., 2011; Pan et al., 2014). Similarly, in *S. miltiorrhiza* hairy roots, treatment with UV-B (0.4 W m^−2^) increased the content of total tanshinones and the transcript levels of *1-deoxy-D-xylulose-5-phosphate synthase* (*SmDXS2*) and *copalyl diphosphate synthase* (*SmCPS*) by 1.5-, 6.2-, and 7.3-fold, respectively, compared with the control (Wang et al., 2016).

Red and blue light are also effective regulators of terpenoid biosynthesis. Generally, red light enhances the accumulation of terpenoids, whereas blue light inhibits terpenoid biosynthesis. Kuo et al. (2015) planted *Aquilaria agallocha* seedlings under different light conditions and found that red light significantly enhanced the contents of cucurbitacin E and I in this species. Similarly, Wang et al. (2018) revealed that red light enhances the production of gypenoside and upregulates the expression of *squalene synthase* (*SS*) and *squalene epoxidase* (*SE*) genes in *Gynostemma pentaphyllum*. Chen et al. (2018) reported that red and blue light irradiation dramatically changes the accumulation of tanshinones in *S. miltiorrhiza* hairy roots; red light treatment upregulated the expression of *SmHMGR*, *SmDXS2*, *SmDXR*, *SmGGPPS*, *SmCPS*, and *CYP76AH1* genes and increased the content of tanshinone II_A_ by 1.4-fold compared with the control, whereas blue light remarkedly suppressed tanshinone II_A_ biosynthesis and downregulated the expression of key tanshinone II_A_ biosynthesis genes. de Carvalho et al. (2020) treated nodal segments of *Dysphania ambrosioides* with different light qualities and showed that blue light inhibited the accumulation of Z-ascaridole in this herb. The effects of red and blue light on terpenoid production are species-specific. For example, both red light and blue light stimulated the biosynthesis of artemisinin and artemisinic acid in *A. annua* but decreased the production of essential oils in *Melissa officinalis* (Chen, 2017; Zhang et al., 2018b).

### Alkaloids

UV-B is an effective elicitor of alkaloid production and has been confirmed to promote the biosynthesis of several kinds of alkaloids in medicinal plants (Table 1; Peebles et al., 2009; Akula and Ravishankar, 2011). Takshak and Agrawal (2015) revealed that supplementary UV-B (ambient +0.042 Wm^−2^) treatment increased the alkaloid content of leaves and roots of *C. forskohlii*. In *Clematis terniflora*, UV-B irradiation (1.208 W m^−2^), followed by dark incubation, increased the content of indole alkaloid (6-hydroxyl-1H-indol-3-yl) carboxylic acid methyl ester by 7-fold (Gao et al., 2016). Similarly, 6 h of UV-B irradiation (1.208 W m^−2^), followed by dark incubation, significantly enhanced the contents of protoberberine alkaloids, including berberine, jateorhizine, palmatine, and columbamine, in *Mahonia bealei* leaves (Zhang et al., 2014). Ramani and Jayabaskaran (2008) showed that treatment of the suspension cultures of *Catharanthus roseus* with UV-B for 5 min increased the contents of catharanthine and vindoline by 3- and 12-fold, respectively. Many key enzyme-encoding genes in the alkaloid biosynthetic pathways are UV-B inducible (Figures 3B,C; Takshak and Agrawal, 2019). For example, Zhu et al. (2015) exposed *C. roseus* to binary stress (enhanced UV-B followed by dark incubation), and found that most of structural genes in the alkaloid biosynthetic pathways were upregulated, among which *10-hydroxygeranioloxidoreductase* (*10-HGO*), *tabersonine 16-hydroxylase* (*T16H*), and *strictosidine synthase* (*STR*) genes were upregulated by approximately 2-, 4-, and 4-fold, respectively, compared with the control. Gao et al. (2016) reported that UV-B irradiation remarkedly stimulated the expression of upstream genes in the indole alkaloid biosynthetic pathways in *C. terniflora*. The expression of key genes in the tropane alkaloid biosynthetic pathway is also affected by UV-B. In *Anisodus luridus* hairy roots treated with UV-B (90 W m^−2^) for 24 h, transcript levels of *putrescine N-methyltransferase* (*PMT*), *tropinone reductase I* (*TRI*), *cytochrome P450 80F1* (*CYP80F1*), and *hyoscyamine 6b-hydroxylase* (*H6H*) genes were increased by 10-, 52-, 16-, and 9-fold, respectively, compared with the control (Qin et al., 2014).

Similar to UV-B, blue and red light are also reported to stimulate the accumulation of alkaloids as well as the transcript levels of related genes (Figures 3B,C; Takshak and Agrawal, 2019). Li et al. (2021) revealed that blue light irradiation (100 μmol m^−2^ s^−1^, 30 d) dramatically increased the production of galanthamine, lycorine, and lycoramine as well as the expression of *norbelladine synthase* (*NBS*), *OMT*, and *CYP96T* in *Lycoris longituba*. Liu et al. (2015) treated *Camptotheca acuminata* seedlings with different light conditions and showed that blue light (1,200±50 μmol m^−2^ s^−1^, 45 d) promoted the camptothecin content, tryptophan decarboxylase (TDC) and tryptophan synthase (TSB) activities, and *TSB*, *TDC1*, and *TDC2* transcript levels. Matsuura et al. (2016) reported that blue light is more beneficial for the production of the monoterpene indole alkaloid N,β-D-glucopyranosyl vincosamide than other light qualities in *Psychotria leiocarp*. Red light is considered as another regulator of alkaloid biosynthesis, and its function is dependent on phytochromes as well as secondary messengers (G protein and CaM; Aerts and De Luca, 1992; Wang et al., 2010). In *C. roseus*, red light induces vindoline production by increasing the expression of the transcription factor gene *GATA1* and vindoline pathway genes *T16H2*, *tabersonine-3-oxygenase* (*T3O*), *tabersonine-3-reductase* (*T3R*), *desacetoxyvindoline-4-hydroxylase* (*D4H*), and *DAT*. In the dark, PIF1 suppresses the expression of the abovementioned genes, which dramatically decreases the accumulation of vindoline (Liu et al., 2019). Similarly, red light treatment (150 μmol m^−2^ s^−1^) significantly enhanced the concentration and yield of vindoline and catharanthine in *C. roseus* seedlings (Ohashi et al., 2013).

## Light Intensity

Optimal light conditions required for growth and development differ among different medicinal plant species. According to their sunlight intensity requirements, medicinal plants are classified into three types: heliophytes, sciophytes, and intermediates. Similar to other physiological processes, the accumulation of SMs in medicinal plants is significantly affected by light intensity (Chen et al., 2017; Li et al., 2020). Generally, high light intensity promotes SM production in heliophytes, such as *Ginkgo biloba* (Xu et al., 2014), *Lonicera japonica* (Fang et al., 2020), *Tabernaemontana pachysiphon* (Hoft et al., 1996), and *Andrographis paniculata* (Saravanan et al., 2008), while low light intensity promotes SM production in sciophytes, such as *Glechoma longituba* (Zhang et al., 2015), *Changium smyrnioides* (Wang et al., 2017), *Polygonum minus* (Mohd Yusof et al., 2021), and *Panax ginseng* (Jung et al., 2020). Concentration and yield are two important parameters that should be considered for SM production in medicinal plants. For example, Li et al. (2018a) reported that 30 and 50% sunlight are better light conditions than 10 and 100% sunlight, for total alkaloid production in *Mahonia breviracema*, as the former light intensities result in higher biomass. Similarly, in *G. longituba*, the concentration and yield of ursolic acid and oleanolic acid were stimulated under 33% sunlight and 16% sunlight, respectively, compared with other light intensities (Zhang et al., 2015).

Light intensity also affects the chemical composition of medicinal plants. For instance, Xu et al. (2020) fond that moderate shade (38.8% of the control) promoted the accumulation of C_6_C_1_- and C_6_C_3_-type phenolics, while severe shading (16.9% of the control) stimulated the accumulation of C_6_C_3_C_6_-type phenolics in *Eleutherococcus senticosus*. Santos Lazzarini et al. (2018) treated *Lippia gracilis* with different light intensities and showed that the chemical composition of seedlings in the 26 μmol m^−2^ s^−1^ group was more complex, with more monoterpene hydrocarbons and less aromatic monoterpenes, than those in other treated groups. In *M. breviracema*, light intensity had different effects on the production of alkaloids and essential oils; alkaloid content was significantly enhanced in the 30 and 50% sunlight groups, while the accumulation of essential oils increased linearly with the increase in light intensity, reaching maximum levels in the full (100%) sunlight group (Li et al., 2018a).

## Photoperiod

Photoperiod is one of the critical environmental factors that regulate the growth and development of medicinal plants and is often closely related to other environmental factors, such as latitude, slope direction, and seasonal changes (Liebelt et al., 2019). Depending on the day length or amount of light required for flowering, medicinal plants are classified into long-day, short-day, and intermediate-day plants (Huang and Liu, 2015). Different plant species adapt to changes in the photoperiod through various physiological modifications, one of which is by altering the accumulation of SMs (Moyo et al., 2014; Zahir et al., 2014; Liebelt et al., 2019).

In many medicinal plants, photoperiod promotes the accumulation of SMs. For example, Fazal et al. (2016b) optimized the conditions required for *P. vulgaris* suspension culture and found that the biomass and SM content were higher under 18-h light/12-h dark (18L/12D), 16L/14D, and 14L/16D photoperiods compared with the control (16L/8D). Kumar et al. (2020) found that *Basella rubra* callus cultures under the 16:8 h photoperiod produced the highest amount of phenolics compared with those under continuous light and continuous dark conditions. Wu et al. (2007) studied the effects of photoperiod on the growth and caffeic acid derivative content of the adventitious root cultures of *Echinacea purpurea*, and found that the accumulation of caffeic acid derivatives was optimum in cultures grown under 3-h light/21-h dark conditions. There are also many studies which report that continuous light or continuous dark is more efficiency to stimulate the bioactive compounds biosynthesis compared with photoperiod in some medicinal plants. For instance, de Castro et al. (2019) found that 24-h d^−1^ light was the best light condition for enhancing the growth and essential oil content of *Lippia alba* seedlings grown *in vitro*. Anjum et al. (2017) treated the cell cultures of *Linum usitatissimum* with different photoperiods, and showed that continuous dark conditions led to the greatest increase in total phenolics (116.85 mg L^−1^) and total flavonoids (37.05 mg L^−1^). Photoperiod also affects the chemical composition of medicinal plants. Tusevski et al. (2013) found that hairy roots of *Hypericum perforatum* cultured under 16-h light/8-h dark photoperiod showed *de novo* biosynthesis of two phenolic acids, three flavonol glycosides, and five xanthones compared those cultured under continuous dark. Fonseca et al. (2006) revealed that dark incubation decreased the content of parthenolide and increased the content of total phenolics in *Tanacetum parthenium*, while photoperiod showed an opposite effect on the accumulation of these compounds.

## Summary and Perspectives

**Figure 4:**
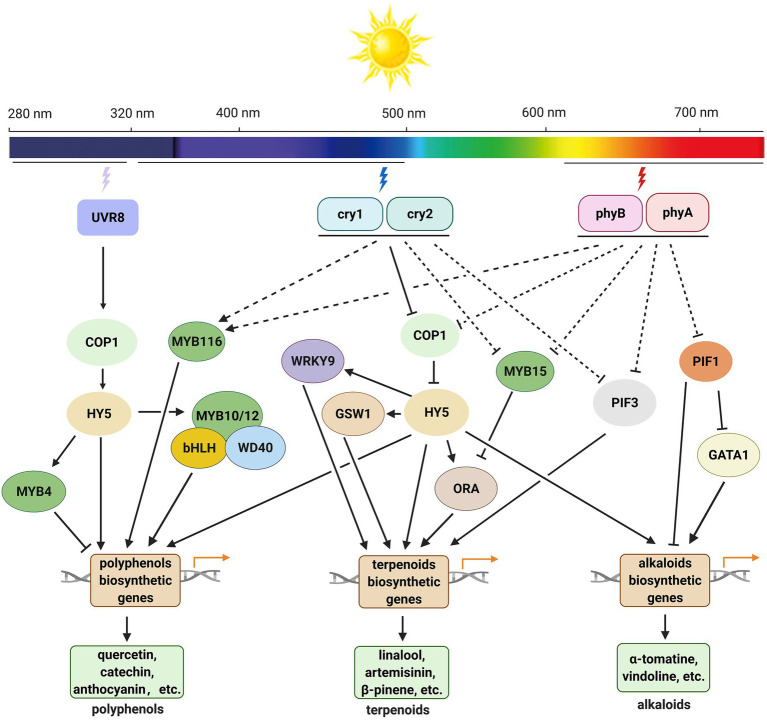
Working model for light-mediated biosynthesis of polyphenols, terpenoids, and alkaloids in medicinal plants. Upon UV-B radiation, the UVR8 homodimer undergoes monomerization and interacts with COP1, which increases COP1 stability and induces *HY5* expression. HY5 regulates the expression of genes encoding transcription factors and/or key enzymes involved in the biosynthesis of SMs and affects the accumulation of SMs under UV-B. Under blue and red light, cryptochromes (cry1 and cry 2) and phytochromes (phyA and phyB) inhibit the E3 ligase activity of COP1, HY5 accumulates in nucleus and promotes the expression of transcription factor and/or key enzyme genes in the related biosynthetic pathways of SMs. Phytochromes may also affect the accumulation of alkaloids by mediating the function of PIF1 and PIF3 in some species. COP1, CONSTITUTIVELY PHOTOMORPHOGENIC1; cry1 and cry2, cryptochrome 1 and cryptochrome 2; GATA1, GATA-type transcription factor 1; GSW1, GLANDULAR TRICHOME-SPECIFIC WRKY 1; HY5, LONG HYPOCOTYL 5; ORA, AP2/ERF type transcription factor; phyA and phyB, phytochrome A and phytochrome B; PIF1, phytochrome interacting factor 1; PIF3, phytochrome interacting factor 3; UVR8, UV RESISTANCE LOCUS 8.

In this review, we summarized the regulatory roles of light quality, light intensity, and photoperiod in SM accumulation in medicinal plants and the known mechanisms underlying these roles (Figure 4). Generally, when plants are exposed to UV-B radiation, the UVR8 homodimer undergoes monomerization and interacts with CONSTITUTIVELY PHOTOMORPHOGENIC1 (COP1), which increases COP1 stability and induces *LONG HYPOCOTYL 5* (*HY5*) expression. HY5 is a core transcription factor in the light signaling pathway that regulates expression of genes encoding transcription factors or key enzymes involved in the biosynthesis of SMs, to affect the accumulation of SMs under UV-B (Gangappa and Botto, 2016; Liu et al., 2018b; Yang et al., 2018). Phytochromes (phyA and phyB) and cryptochromes (cry1 and cry2) regulate the accumulation of SMs in a different way (Hemm et al., 2004; Chen et al., 2006; Li et al., 2014; Fu et al., 2021a). These photoreceptors interact with COP1 or SUPPRESSOR OF PHYA-105 (SPA) in a light-dependent manner and inhibit the E3 ubiquitin ligase activity of the COP1–SPA complex. Under dark conditions, COP1 enters the nucleus and mediates the degradation of the HY5 protein through the 26S proteasome. Under blue, red, and far-red light conditions, HY5 functions its roles normally and promotes the production of SMs. In *A. annua*, HY5 binds to the promoters of some transcription factor genes, such as *AaWRKY9*, *GLANDULAR TRICHOME-SPECIFIC WRKY 1* (*AaGSW1*), and *AaORA*, to upregulates their expression. These transcription factors then stimulate the expression of related biosynthetic genes and enhance the content of artemisinin (Hao et al., 2019; Fu et al., 2021b). HY5 can also affect the artemisinin biosynthesis by directly regulate the expression of biosynthetic genes in *A. annua* (Zhou et al., 2015). Phytochrome Interacting Factors (PIFs) also mediate the biosynthesis of SMs in many plant species. In *C. roseus*, under dark conditions, PIF1 binds to the promoters of *deacetylvindoline-4-O-acetyltransferase* (*DAT*) and GATA-type transcription factor gene *GATA1*, thus repressing the expression of target genes and decreasing the content of vindoline. Under red light, PIF1 is degraded, which stimulates the accumulation of vindoline (Liu et al., 2019). PIF3 upregulates the expression of *ADS*, *CYP71AV1*, *artemisinic aldehyde Δ11(13) reductase* (*DBR2*), and *aldehyde dehydrogenase 1* (*ALDH1*), and dramatically increases the content of artemisinin under light in *A. annua* (Zhang et al., 2019c). AaMYB15 is reported as a negative regulator of artemisinin, it binds to the promoter of *AaORA* and inhibits the biosynthesis of artemisinin under light in *A. annua* (Wu et al., 2021).

As an indispensable environmental factor, light affects the contents and yields of almost all kinds of phytochemicals in medicinal plants. The responses of plants to light are species-specific, and the effects of light on SM biosynthesis are distinct among different species. Moderate UV-B and blue light irradiation improve the accumulation of phytochemicals in many medicinal plants. Compared with UV-B and blue light, red light promotes the growth and development of some medicinal plants and therefore is more efficient in enhancing the yield of target compounds. In nature, light intensity and photoperiod often act together with other environmental factors, either synergistically or antagonistically, to regulate SM biosynthesis in medicinal plants. The regulatory mechanisms underlying the effects of light intensity and photoperiod on SM biosynthesis have not yet been fully elucidated.

With the increasing demand for natural bioactive compounds, many environmentally controlled systems with artificial light sources have been used for the cultivation of medicinal plants. Light conditions optimal for SM production in different plant species have been determined, and light-responsive genes involved in the corresponding biosynthetic pathways have been characterized. However, the intact light signaling pathways in almost all medicinal plants remain unclear. Besides, the regulatory roles of light in plants are complex. Understanding how light systematically regulates the SM content and growth of medicinal plants, simultaneously affects the content and yield of target compounds is a challenge that should be tackled in the future.

## Author Contributions

SZ, DY, and YW conceptualized and designed the work, collected, analyzed, and interpreted the data, and drafted the manuscript. LZ and HZ collected the data and contributed to critical revision of the manuscript. LQ, YZ, and HZ approved the final version to be published. All authors contributed to the article and approved the submitted version.

## Funding

This work was financially supported by the National Natural Science Foundation of China (31700257), Key project at central government level: The ability establishment of sustainable use for valuable Chinese medicine resources (2060302), the Zhejiang Provincial Natural Science Foundation of China (LR21H280002).

## Conflict of Interest

The authors declare that the research was conducted in the absence of any commercial or financial relationships that could be construed as a potential conflict of interest.

## Publisher’s Note

All claims expressed in this article are solely those of the authors and do not necessarily represent those of their affiliated organizations, or those of the publisher, the editors and the reviewers. Any product that may be evaluated in this article, or claim that may be made by its manufacturer, is not guaranteed or endorsed by the publisher.
